# Nation-wide mapping of tree-level aboveground carbon stocks in Rwanda

**DOI:** 10.1038/s41558-022-01544-w

**Published:** 2022-12-22

**Authors:** Maurice Mugabowindekwe, Martin Brandt, Jérôme Chave, Florian Reiner, David L. Skole, Ankit Kariryaa, Christian Igel, Pierre Hiernaux, Philippe Ciais, Ole Mertz, Xiaoye Tong, Sizhuo Li, Gaspard Rwanyiziri, Thaulin Dushimiyimana, Alain Ndoli, Valens Uwizeyimana, Jens-Peter Barnekow Lillesø, Fabian Gieseke, Compton J. Tucker, Sassan Saatchi, Rasmus Fensholt

**Affiliations:** 1 Department of Geosciences and Natural Resource Management, University of Copenhagen, Copenhagen, Denmark; 2 Centre for Geographic Information Systems and Remote Sensing, College of Science and Technology, University of Rwanda, Kigali, Rwanda; 3 Laboratoire Evolution et Diversité Biologique, CNRS, UPS, IRD, Université Paul Sabatier, Toulouse, France; 4 Global Observatory for Ecosystem Services, Department of Forestry, Michigan State University, East Lansing, MI USA; 5 Department of Computer Science, University of Copenhagen, Copenhagen, Denmark; 6 Pastoralisme Conseil, Caylus, France; 7 Laboratoire des Sciences du Climat et de l’Environnement, CEA/CNRS/UVSQ/Université Paris Saclay, Gif-sur-Yvette, France; 8 Université Paris Saclay, Gif-sur-Yvette, France; 9 Department of Geography and Urban Planning, College of Science and Technology, University of Rwanda, Kigali, Rwanda; 10 International Union for Conservation of Nature—Eastern and Southern Africa Region, Kigali, Rwanda; 11 General Directorate of Land, Water, and Forestry, Ministry of Environment, Kigali, Rwanda; 12 Division of Forest, Nature and Landscape, Department of Earth and Environmental Sciences, KU Leuven, Leuven, Belgium; 13 Department of Information Systems, University of Münster, Münster, Germany; 14 Earth Sciences Division, NASA Goddard Space Flight Center, Greenbelt, MD USA; 15 Jet Propulsion Laboratory, California Institute of Technology, Pasadena, CA USA

**Keywords:** Forest ecology, Climate-change ecology

## Abstract

Trees sustain livelihoods and mitigate climate change but a predominance of trees outside forests and limited resources make it difficult for many tropical countries to conduct automated nation-wide inventories. Here, we propose an approach to map the carbon stock of each individual overstory tree at the national scale of Rwanda using aerial imagery from 2008 and deep learning. We show that 72% of the mapped trees are located in farmlands and savannas and 17% in plantations, accounting for 48.6% of the national aboveground carbon stocks. Natural forests cover 11% of the total tree count and 51.4% of the national carbon stocks, with an overall carbon stock uncertainty of 16.9%. The mapping of all trees allows partitioning to any landscapes classification and is urgently needed for effective planning and monitoring of restoration activities as well as for optimization of carbon sequestration, biodiversity and economic benefits of trees.

## Abstract (web summary)

The authors conduct a national inventory on individual tree carbon stocks in Rwanda using aerial imagery and deep learning. Most mapped trees are located in farmlands; new methods allow partitioning to any landscape categories, effective planning and optimization of carbon sequestration and the economic benefits of trees.

## Main

Trees both inside and outside forests are important features of many ecosystems^1^. Individual tree traits are key determinants of ecosystem services including carbon storage, climate regulation and fuel wood^2,3^. Many countries regularly evaluate, quantify and monitor forests using field inventories as part of national forest monitoring systems^4–6^. Forest inventories are the backbone to measurement, reporting and validation (MRV) for climate change mitigation initiatives such as national level REDD+ (reducing emissions from deforestation and forest degradation)^5,6^, Sustainable Development Goals (SDGs) especially SDG 15 (ref. ^7^), the Paris Agreement^8^ and the Bonn Challenge^9^. In many northern countries, sample-based forest inventories are often accompanied by airborne LiDAR campaigns, providing detailed information on the carbon stocks of forests. However, field inventories and LiDAR campaigns are expensive and labour intensive^4^, resulting in trade-offs between accuracy, reproducibility and the frequency of reporting. In many tropical countries, financial and human resource constraints limit the coverage and frequency of the field inventories.

This is particularly problematic for many African countries, where submetre and country-scale LiDAR data are not available across a variety of landscape types, ranging from savannas, woodlands, subhumid and humid forests, to highly fragmented, small-scale agro-ecosystem mosaics^10,11^. This complexity makes it difficult to scale from sparsely sampled plots to the national scale. Indeed, many of these landscapes are dominated by non-forest trees which are very difficult to map with traditional methods^12,13^.

Inventories and existing large-scale tree cover maps often omit an accounting of trees growing outside forests, which is related to differences in forest definitions, mapping techniques and the complexity of the environment^14–16^. This leads to incomplete censuses of trees and their related benefits and services at a national scale^17,18^. More specifically, these inaccuracies aggravate the existing uncertainties in estimates of both national carbon stocks and emission reference levels and may confound the relative contributions of emissions attributed to forest degradation or deforestation^18–20^. In turn, these inaccuracies in tree mapping complicate adequate natural resource management, climate change decision-making and policy formulation^21^. These challenges are especially notable in the tropics, where many landscape restoration projects have been initiated without fully functional monitoring systems in place^22,23^.

In Africa, the rate of tree cover loss in natural forests and projected biomass losses from climate change have prompted both policy and economic initiatives for the restoration of tree-dominated landscapes^24–26^. Ongoing initiatives include the African Forest Landscape Restoration Initiative (AFR100), with more than 30 African governments making commitments to restore at least 100 million hectares of land across the continent by 2030 (ref. ^22^), the Africa Low Emissions Development Strategies^27^ and the Great Green Wall initiative^28^. However, there is currently no accurate and regularly updated monitoring platform to track the progress and biophysical impact of these initiatives^22,29^. Here, we propose an approach for rapidly and accurately mapping individual trees and quantifying their carbon stocks at national scale. We also illustrate how the aforementioned challenges can be addressed with efficient new monitoring tools, using Rwanda as a demonstration. The country is a signatory to most of the above-mentioned global climate mitigation initiatives and regularly reports on their implementation. Rwanda targeted at least 30% of the country to be covered by forests by the year 2020 (ref. ^30^), which was achieved in 2019 (ref. ^31^) and, under the Bonn Challenge, the country has also committed to restore 81% of the country’s surface area by 2030 (refs, ^31,32^). Moreover, Rwanda represents a great example of a place with contrasting landscape types, the full range and variety of tree-based systems and a rich mixture of land uses: drylands dominated by savannas and pastureland, plateaus dominated by agriculture and humid highlands dominated by natural forests and protected areas, including tropical montane rainforest^33^.

Recently, it was demonstrated that advanced machine learning techniques can map individual trees over large dryland areas^17^. However, the analysis was limited to isolated trees in savannas excluding small trees with a crown area <3 m^2^ and did not cover other complex and heterogeneous ecosystems such as woodlands and forests. Here, we use aerial images and map both crown size and carbon stock of each individual overstory tree in Rwanda, regardless of ecosystem type. We define trees as woody plants visible from above and with a crown size of at least 0.25 m^2^ and a visible shadow. We also show the applicability of the model trained in Rwanda in other African countries using satellite imagery. This study suggests a rapid, reproducible and highly accurate way to upscale field inventory data collected at the level of individual trees to the entire country. This will allow tree inventory reports to be of unprecedented accuracy and can support MRV of climate change mitigation initiatives.

## Mapping individual trees at national scale

**Fig. 1:**
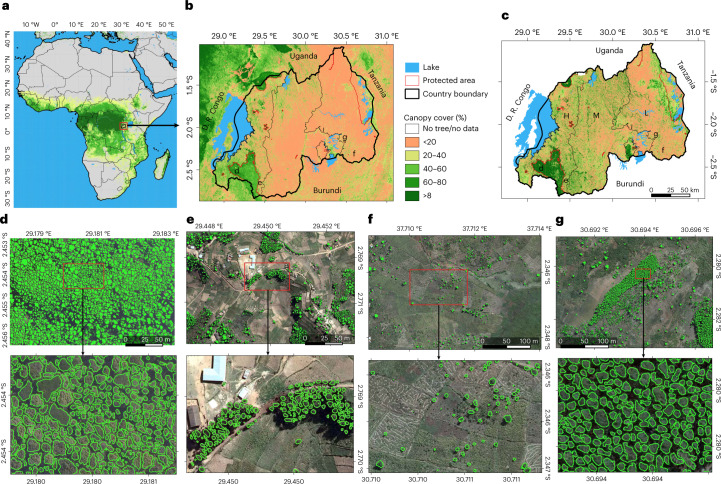
Mapping of individual trees inside and outside of forests in Rwanda. **a**,**b**, Tree cover from a previously published global tree cover map using Landsat data in Africa (**a**) and in Rwanda (**b**). Global tree cover data in **a** and **b** from ref. ^15^. **c**, Country-wide tree cover estimated by deep learning from 0.25 m resolution aerial imagery from 2008 (L, lowlands; M, midlands; H, highlands). Labels **d**–**g** in **a** and **b** show location of expanded panels. **d**–**g**, Examples of individual tree crown mapping in tropical montane rainforest, note that artificial gaps were filled during a postprocessing step (Methods) (**d**); *Eucalyptus* plantations (**e**); farmlands (**f**); and *Pinus* plantations (**g**). An example of previously published manual forest area delineations is shown in Extended Data Fig. 1. Boundary shapefiles of Rwanda in **b** and **c** from https://geodata.rw/portal/home/. Panel **e** adapted with permission from ref. ^50^, Springer Nature Limited. Credit: photographs in **d**–**g**, Swedesurvey.

**Extended Data Fig. 1:**
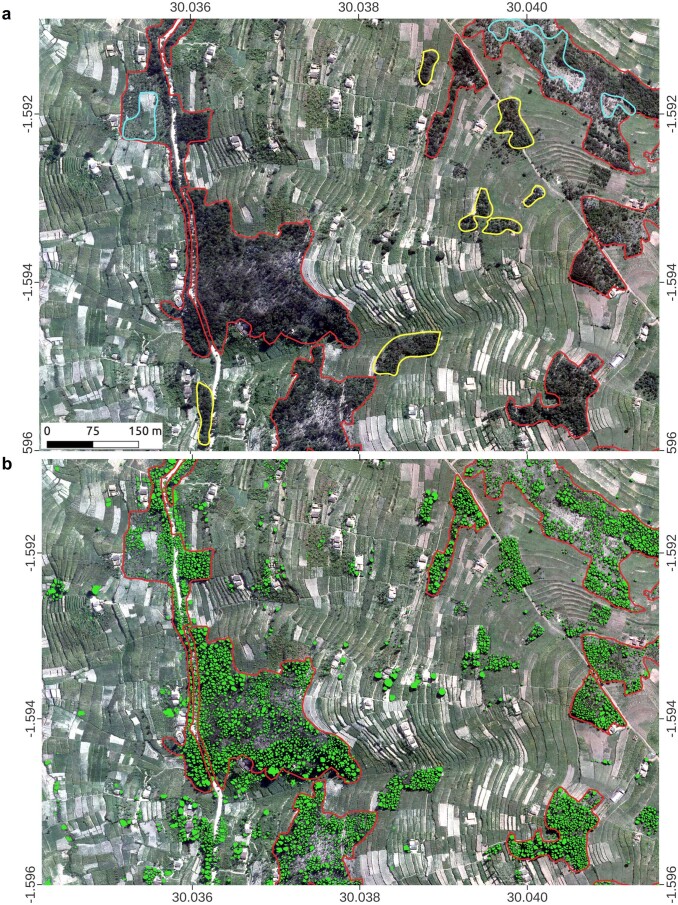
An example showing previous manual forest delineations. The red lines are manually labelled forest boundaries from the Rwanda national forest map of 2012 (ref. ^35^). **a**, Several patches which would qualify for the used forest definition were missed by the manual delineation (yellow lines). Also, some bare areas inside forest patches (cyan line) were considered as part of forests. **b**, Our results are shown in green, highlighting the improvements over the previous method. Shapefiles for national forest cover map from RFA. Credit: background photographs, Swedesurvey.

**Extended Data Fig. 2:**
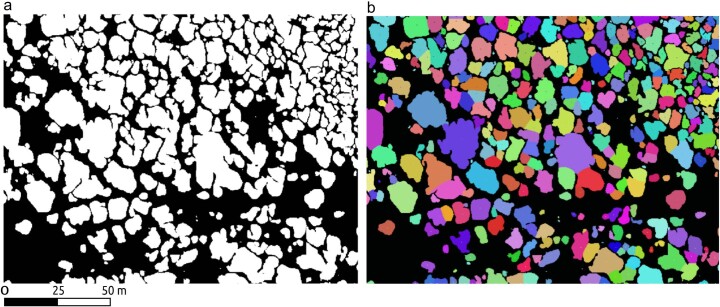
Examples from the postprocessing algorithm separating clumped trees. **a**, Example of predicted tree crowns in the rainforest, with many trees being predicted as clumped objects. **b**, Results after the postprocessing algorithm separated the clumped crowns. The algorithm first finds the object centres, and then relabels the image based on weighted distances to the identified centres. Thus, blobs containing multiple centres are divided into various instances accordingly. The algorithm adds a large penalty for not-in-sight centres, and additionally performs a majority filtering to remove the lone-pixels.

Aerial images with a spatial resolution of 25 × 25 cm^2^ were acquired in 2008 covering the entire country^34^ (Extended Data Fig. 1). A deep-learning model was trained using 97,574 hand-labelled tree crowns and then used to map 355,268,345 trees with crown size >0.25 m^2^ (excluding understory). The crown area threshold was set on the basis of visual inspection of the images, as trees of this size are still clearly visible (Fig. 1). Clumped crowns were separated using a postprocessing method that determines the crown centres in the predictions, assuming that tree crowns have round shapes. The method then relabels the crown predictions on the basis of weighted distances to the identified crown centres (Methods; Extended Data Fig. 2). Crown area and count predictions were validated with an independent test dataset and field data (Methods).

**Fig. 2:**
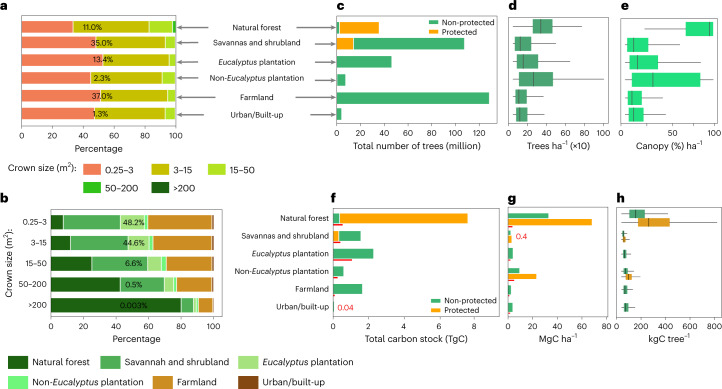
Tree counts, crown areas and carbon stocks for different land cover/use types. **a**, Percentage covered by different crown sizes in each land cover/use type. The percentage number shows the contribution of the land cover/use type to the total area. Crown sizes >200 m^2^ comprise only 0.003% of the total tree count, making the class barely visible. **b**, Percentage covered by each land cover/use type in different crown size categories. **c**, Total count of trees by land cover/use. **d**, Boxplot showing the average number of trees per hectare by land cover/use type. **e**, Same as **d** but for canopy cover. **f**, Total estimated carbon stock per land cover/use type (see Methods for details on uncertainties and error propagation). **g**, Barplot showing the average carbon density per hectare per land cover/use. **h**, Boxplots showing the average carbon stock per tree per land cover/use. Number of trees = 355,268,345 (for **c**–**h**, *n* = 39,347,302 for natural forest; 123,381,245 for savannah and shrubland; 47,921,438 for *Eucalyptus* plantation; 8,296,494 for non-*Eucalyptus* plantation; 131,822,508 for farmland; and 4,499,358 for urban/built-up). In boxplots in **d**, **e** and **h** (from left to right), the start of the horizontal line represents the minimum value, vertical lines represent first quartile, median and third quartile values, respectively, and the end of the horizontal line represents the maximum value. The red lines in **f** and **g** are the error bars presented as absolute difference between the final carbon stock predictions and the NFI data (Extended Data Table 1). The overall uncertainty at national scale is 16.9% (Methods; Extended Data Table 1b).

Following a manual delineation, which is based on the same aerial images as used here and which includes forest patches down to a size of 0.25 ha (ref. ^35^; Extended Data Fig. 1), we stratified the landscapes into broad classes that can be found in most African countries: natural forest (including both large rainforest trees in the Nyungwe park in the southwest of Rwanda and smaller trees in the volcanoes park in the northwest), *Eucalyptus* plantations (excluding isolated *Eucalyptus* trees in farmlands), non-*Eucalyptus* tree plantations, farmlands, urban and built-up areas, as well as savannas and shrublands (Methods). We further subdivided each class in protected and non-protected areas. Overall, our results show a dominance of trees with small crown sizes of 0.25–3 m^2^, which account for 48.2% of the mapped trees, followed by trees with crown sizes of 3–15 m^2^, which account for 44.6% of the trees. Related to our land stratification, these two crown size ranges are dominant in farmlands and *Eucalyptus* plantations (Fig. 2a,b). Trees of the largest crown size class (crown sizes >200 m^2^) are very scarce and mainly found in natural forests which dominate areas under protection.

We show that only 11% of the mapped trees are located inside natural forests, whereas most trees are located in farmland (37%). Specifically, natural forests have 39.3 million trees, with a median density of 298 (s.d. 159) trees ha^−1^ and a median canopy cover of 96.0% (s.d. 31.7). Farmlands have a median tree density of 64 (s.d. 139) trees ha^−1^ and a median canopy cover of 7.6% (s.d. 18.7). *Eucalyptus* plantations account for about 48 million trees (~13.4% of the total mapped trees), a median density of 110 (s.d. 195) trees ha^−1^ and median canopy cover of 13.9% (s.d. 26.3). Here the canopy cover is low because the manually drawn classification of plantation areas also includes bare areas close to plantations (Extended Data Fig. 1). Non-*Eucalyptus* tree plantations have 8.3 million trees (~2.3% of the total mapped trees), a median density of 221 (s.d. 222) trees ha^−1^ and a median canopy cover of 31.5% (s.d. 37.8). Urban and built-up areas have 4.5 million trees (~1.3% of the total mapped trees) and a median density of 74 (s.d. 142) trees ha^−1^. Savannas and shrublands account for 123.3 million trees (~35% of the total mapped trees), with a median density of 100 (s.d. 164) trees ha^−1^ and a median canopy cover of 12.2% (s.d. 23.2). Overall, trees outside of natural forests are about 315.9 million trees of which 41.6% are in farmlands. The quantification of non-forest trees depends on how forests are defined. A total of 89% of the trees in Rwanda do not belong to the classes of natural forests and can thus be considered as trees outside natural forests.

The importance of protected areas for tree density and count is worthwhile to note. Although they cover only 5% of the country, they have 11% of the total mapped trees with the highest median tree density of 298 trees ha^−1^ as well as the highest median canopy cover of 96.3%. Overall, 20.8% of Rwanda was covered by trees (canopy cover) in 2008.

## Carbon stocks estimated for individual trees

**Fig. 3:**
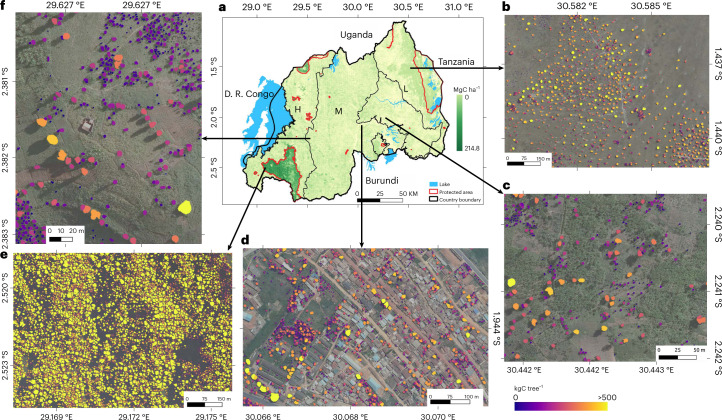
Aboveground carbon stocks at tree level in Rwanda. **a**, Spatial distribution of the estimated carbon stock across the major landscape types. **b**–**f**, Examples of estimated carbon stock per individual tree in wooded savanna (**b**), farmland (**c**), Kigali city (**d**), the tropical montane rainforest in the Nyungwe National Park (**e**) and in tree plantations (**f**). Boundary shapefiles of Rwanda in **a** from https://geodata.rw/portal/home/. Credit: photographs in **b**–**f**, Swedesurvey.

**Extended Data Fig. 3:**
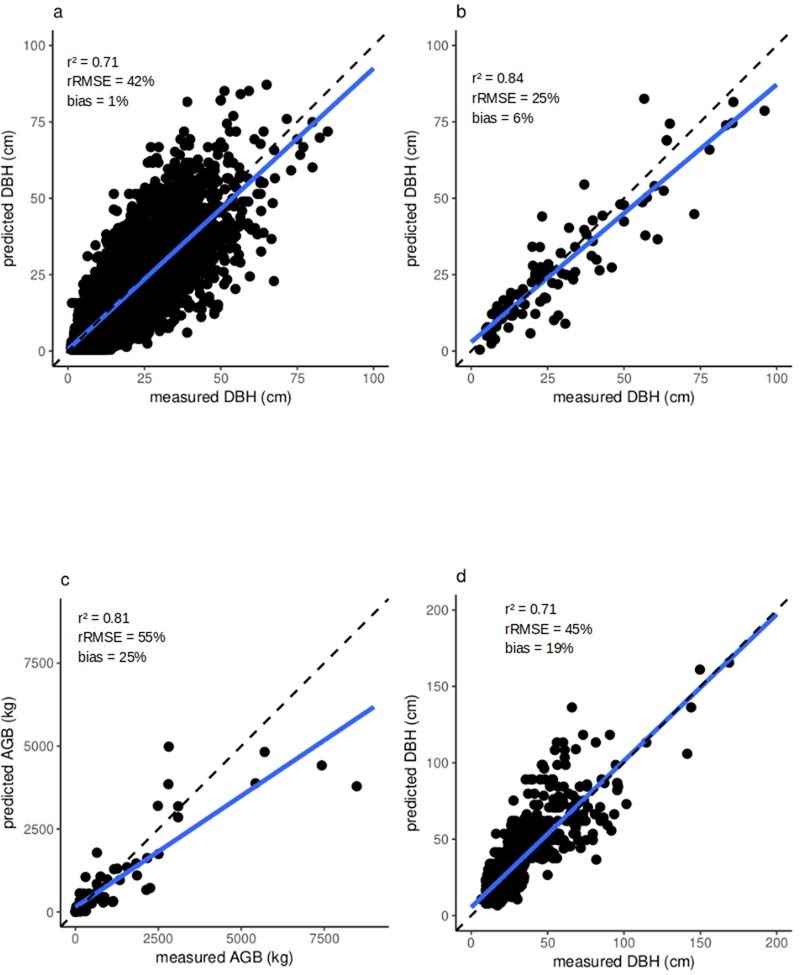
Tree crown allometry. **a**, DBH is predicted from CD using a total of 11,593 samples from a global database^36^. The plot compares the predicted against the measured DBH. **b**, DBH is predicted for an independent dataset of 93 trees from Kenya using the equation derived from (a). **c**, We used an allometric equation from literature^37^ to calculate AGB from the CD predicted DBH shown in (b). Predicted AGB is here compared with destructively measured AGB. **d**, While samples shown in a–c are all outside forests, (d) shows 793 field-measured trees from Rwanda’s natural forest. As in (a), DBH was predicted from CD. The bias was calculated following equation (1).

**Extended Data Fig. 4:**
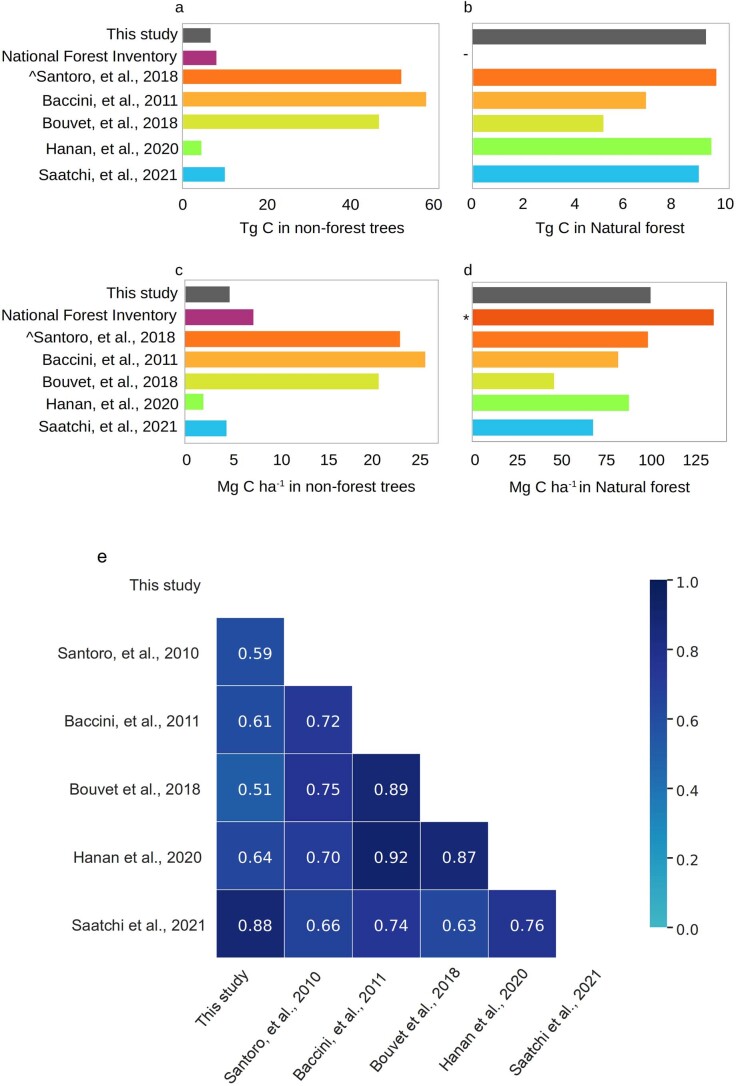
Comparison of aboveground carbon estimations at country scale. **a**, Comparison of predicted total aboveground carbon stocks by this study, NFI and existing global products for non-forest areas. **b**, Same as (a) but for natural forests. **c**, Comparison of predicted aboveground carbon density by this study, NFI, and existing global products for non-forest areas. **d**, Same as (c) but for natural forests. **e**, Correlation matrix showing the two-tailored Pearson correlation for 1×1 km^2^ grids between different biomass datasets. Note that ref. ^45^ does not report values above 85 Mg biomass per hectare (Supplementary Fig. 2), explaining the lower correlation values. P < 0.001 for all correlations. (^ We selected the 2010 version; - Natural forest is not considered as part of the NFI; * ref. ^40^ and ref. ^41^).

**Extended Data Fig. 5:**
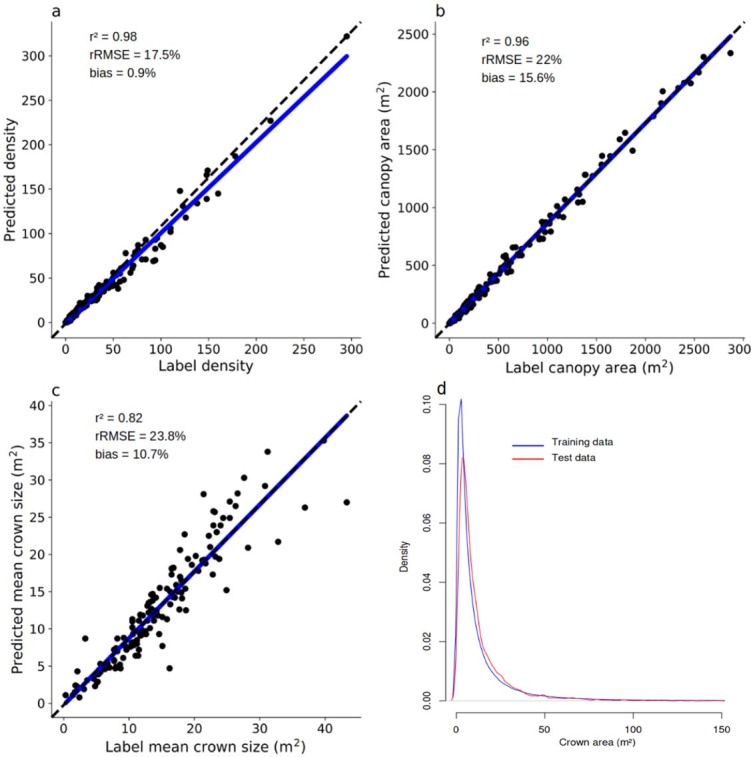
Tree crown model evaluation at plot level. We manually labelled 6,591 trees and compared predictions and labelled trees at plot level. **a**, Tree density, **b**, canopy area, **c**, mean crown size. Each point represents one 25×25 m grid, n = 153. The bias was calculated following equation (2). **d**, Density distribution of the crown areas in the training and test dataset.

**Extended Data Fig. 6:**
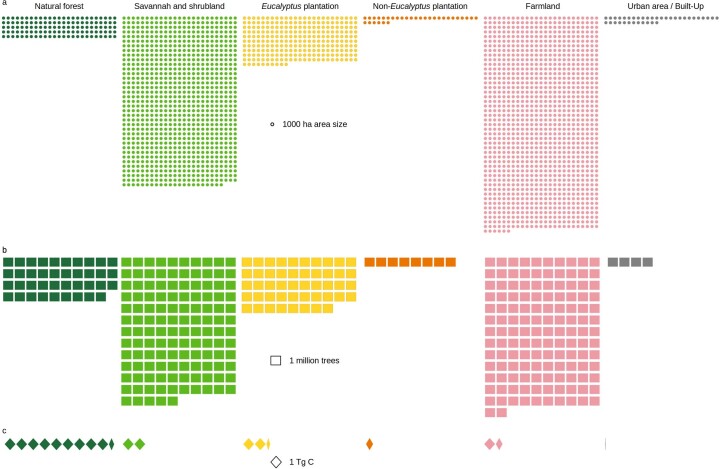
Visual illustration of tree count and carbon stock per land cover type in Rwanda. **a**, Area size (1000 ha) covered by different land cover types, and **b**, tree count per land cover type (million trees), and **c**, carbon stocks per land cover type (TgC). Although natural forests cover a small portion of land, they are home to a remarkable number of trees with the largest C stocks of the country.

We used data from a field campaign in December 2021 and existing databases to estimate the stem diameter from the mapped crown sizes via allometric equations^36–39^ (Methods). We then estimated the aboveground carbon stocks for each tree using existing equations based on stem diameter (Methods; Extended Data Figs. 3–5). We established land cover class-specific relationships and report here the combined results using allometric equations from ref. ^38^ for natural forest; ref. ^39^ for *Eucalyptus* and non-*Eucalyptus* plantations, farmlands and urban and built-up areas; and ref. ^37^ for savannas and shrublands. For each class, we evaluated the aggregated carbon stocks with data from the Rwanda National Forest Inventory (NFI) from 2013/2014 and field data from refs. ^40,41^, covering all land cover classes and serving as a measure of uncertainty which we quantify to 16.9% at national scale (Extended Data Tables 1 and 2). We estimate a total of 14.3 ± 2.8 Tg (± is the uncertainty) of aboveground carbon stocks in trees (Fig. 3, Extended Data Figs. 4 and 6 and Extended Data Tables 1 and 2). For areas outside the natural forests, we estimate 7.0 ± 1.1 TgC, which is 48.6% of the total national aboveground carbon stocks and slightly lower than NFI estimates (8.4 TgC). Farmlands have a total estimate of 3.5 ± 0.2 TgC corresponding to 24.4% of the national aboveground C stock with a C density of 3.0 ± 0.18 (s.d. 3.2) MgC ha^−1^. Urban and built-up areas have 0.1 ± 0.006 TgC corresponding to 1.03% of the national aboveground C stock with a C density of 4.0 ± 0.24 (s.d. 4.0) MgC ha^−1^. *Eucalyptus* plantations comprise a total estimate of 1.1 ± 0.6 TgC corresponding to 7.7% of the national aboveground C stocks, with a C density of 4.2 ± 2.2 (s.d. 4.3) MgC ha^−1^. These low estimates can be explained by sparse tree planting and the regular harvesting keeping the trees young^39,40^. Non-*Eucalyptus* plantations have a total estimate of 0.3 ± 0.16 TgC corresponding to 2.2% of the national aboveground carbon stocks, with a C density of 10.0 ± 5.2 (s.d. 9.9) MgC ha^−1^. Savannas and shrublands have 1.9 ± 0.36 TgC corresponding to 13.2% of the national aboveground C stock with a C density of 2.4 ± 0.45 (s.d. 2.4) MgC ha^−1^. For natural forests, we estimate a median C density of 81 ± 21 MgC ha^−1^ for areas where field data are available (the Nyungwe tropical montane rainforest), which is lower than the field measurements of 121 MgC ha^−1^ (refs. ^40,41^), possibly due to trees from lower layers not visible from above. Overall, we estimate that 51.6% of the total national aboveground carbon stocks are in natural forests with a total estimate of 7.4 ± 2.47 TgC and an overall C density of 62 ± 20 (s.d. 31.2) MgC ha^−1^.

## Scalability of the approach

**Fig. 4:**
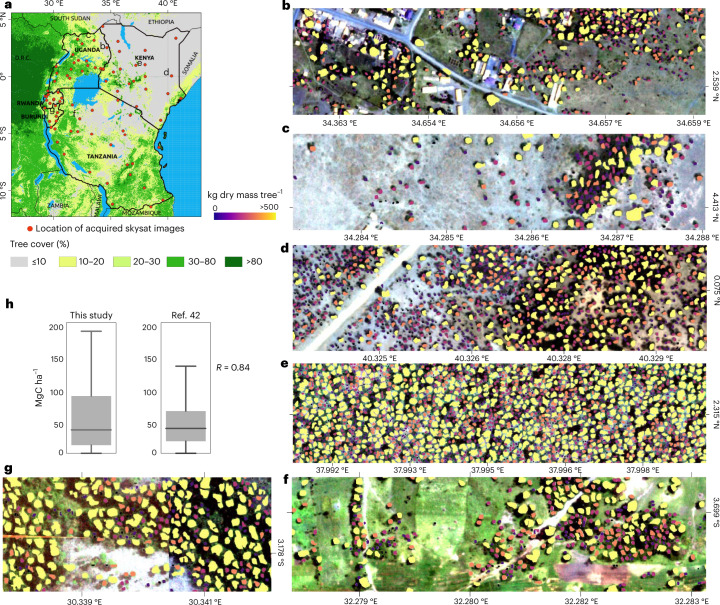
Application beyond Rwanda. **a**, The location of the acquired 83 Skysat images across East Africa. Tree cover data from ref. ^15^. **b**–**g**, Examples of tree-level carbon stock predictions for Uganda (**b**,**c**), Kenya (**d**,**e**), Tanzania (**f**) and Burundi (**g**). **h**, A total of 150 random (*n* = 150) 1 × 1 km^2^ samples were used to aggregate the predicted tree-level carbon stocks, then compare the results to a previously published map^42^ using two-tailed Pearson correlation (*P* < 0.001). The boxplot lines from top to bottom represent the maximum, third quartile, median, first quartile and minimum values. See Extended Data Fig. 8a for more comparisons. Boundary shapefiles of East African countries in **a** from DIVA-GIS. Credit: photographs in **b**–**d**, Skysat.

We acquired 83 Skysat images at 80 cm ground sampling resolution for 2019–2021 for Tanzania, Kenya, Uganda, Burundi and Rwanda and directly applied the model trained with the aerial images from Rwanda (Fig. 4). The tree detection and crown delineation work elsewhere, even beyond Africa (Extended Data Fig. 8). We then compared the predicted carbon density from 150 randomly selected 1 × 1 km^2^ patches with previously published maps of biomass^42–45^. Both the magnitudes of the predictions (Fig. 4b) and the spatial correlation (*R* = 0.84) match well with the most recent map^42^.

Assessments and reporting schemes for tree resources at country scale are limited by poor data quality and ambiguous or inconsistent definitions of land cover and land use types^2,46^. Forests have been defined differently depending on location, measurement protocols and landscape contexts. When forests are specified as continuous tree cover at a predetermined mapping resolution, the underlying definition and the quality of the data product are essential to understand precisely what is being mapped and ambiguities and uncertainties result from different datasets. For instance, at medium spatial resolution >5–10 m, tree cover products may be dominated by continuous clusters of trees of a certain size and may exclude many isolated trees. Also, previous work has overestimated the extent of forests by the inclusion of shrubs and bushes as forestland^47^. Uncertainties in tree cover estimates propagate to estimations of carbon stocks, resulting in large uncertainties in biomass and carbon estimates and maps^48^ (Extended Data Fig. 4 and Extended Data Table 1b).

This analysis presents an assessment of all overstory trees, aside from the assessment of their cover, density or land use. We estimate aboveground carbon stocks at the tree level, not on an aggregate area basis as it has been in most previous carbon stock mapping. This approach is more closely aligned with standard methods and protocols for allometric scaling of carbon from inventory plots. Most allometric scaling from a tree parameter to biomass or carbon involves nonlinear relationships, where the size class distribution of trees matters for the final estimate of landscape carbon stocks. Furthermore, the ability to map all trees makes our assessment independent of forest, tree cover and land use definitions. Reporting carbon stocks on the basis of individual trees would solve important uncertainties caused by definitions, methods, data sources and spatial resolution. Our example shows that even a very detailed manual forest delineation approach missed 38.4% of the isolated trees in Rwanda, which account for 25.5% of the national aboveground carbon stocks. With results as presented here, management and conservation decision-making can be more targeted by discriminating various types of tree systems under specific conditions and in specific locations. Our approach therefore could support management of a broad array of tree-based systems such as agroforestry, as well as catalyse important cross-sector management strategies inclusive of Agriculture, Forestry and other Land Use (AFoLU). A focus on AFoLU provides a way to simultaneously manage both emission reductions and removals of greenhouse gases^49^. Integrating non-forest trees in conservation initiatives expands the scope of climate change mitigation and adaptation efforts by also accounting for trees not included in standard forest assessments^50^. Forests have been an important focal point for land cover change monitoring for decades and progress has been made developing tools and methods applied to forests^1–3^. However, less effort has been given to monitoring trees and carbon stocks for trees outside of forests. This would provide a more robust approach for reducing emissions and increasing removals, making it possible to implement comprehensive national climate change mitigation actions in countries with mostly sparse tree cover. This analysis deploys a complete inventory of trees in AFoLU as a comprehensive approach that could support a more inclusive formulation of national programmes. Moreover, when considering the large number of trees in croplands, it is possible to monitor—and thus include in policies—economically poor rural communities in marginal landscapes that are particularly vulnerable to climate change. Quantitative data as presented here have the potential to link both mitigation and adaptation in a single framework. Many of the non-forest tree systems have direct connections to livelihoods and therefore are also important for economic development, poverty alleviation and climate change resilience and adaptation. Through innovations in landscape management practices using tree-based systems and associated monitoring and reporting systems, millions of farmers could be important participants in climate change mitigation actions, while also enhancing their livelihoods at the same time. By bringing a broad array of tree-based systems into carbon monitoring platforms, there is an opportunity to increase the engagement of countries with low tree densities in climate change mitigation.

The aerial images and NFI data used here have a high quality and cover the entire country, which is a ‘best-case’ scenario that can hardly be met by countries with limited resources and heterogeneous landscapes. However, we have demonstrated that our model trained on aerial images in Rwanda can be directly applied on images from Skysat satellites, which can be obtained for a reasonable price for monitoring restoration projects.

We acknowledge that a satellite- or aerial image-based approach cannot resolve the full range of crown sizes because small trees are shaded out by large trees, and also the conversion of crown area to carbon stocks includes uncertainty^36^ (Methods; Extended Data Table 1b). To reduce uncertainty, future versions of the proposed method should include more localized field inventory databases that allow for more local biome and species-specific wood densities and allometric conversions, as well as integrate tree height estimations from field and airborne LiDAR surveys. Ideally, national inventories are coupled with results such as those presented here to identify systematic bias and optimize the upscaling from field plot information to country scale. If limited funds are available, low-cost imagery from PlanetScope or Sentinel-2 may be trained with tree-level data such as provided here, which would also allow a deeper temporal dimension.

Having the capability of mapping tree-level carbon stocks is important to a range of applications including monitoring of forest landscape restoration, tree plantation survival rate, forest demography: dynamics of mortality and recruitment, tree-dominated land ownership, payments for ecosystem services, issuance of concession permits and tracking compliance, among other benefits. Therefore, we emphasize the inclusion of funding for regular high-resolution imagery along with localized field inventory databases in development packages. We also highlight the relevance of all trees for conservation and protection efforts and encourage that trees outside forests are considered as equally important as trees in forests.

## Methods

### Aerial images

We use publicly available aerial images of Rwanda at 0.25 × 0.25 m^2^ resolution, collected in June–August of 2008 and 2009. The images were acquired from 3,000 m altitude above ground level, originally with a mean ground resolution of 0.22 × 0.22 m^2^ pixel size then resampled to 0.25 × 0.25 m^2^, using a Vexcel UltraCam-X aerial digital photography camera^34^. The images exhibit a red, green and blue band stored under 8 bit unsigned integer format. The aerial images cover 96% of the country and the remaining 4% was filled with satellite images from WorldView-2, Ikonos, Spot and QuickBird satellite sensors which are part of the publicly available dataset.

### Environmental data

**Extended Data Fig. 7:**
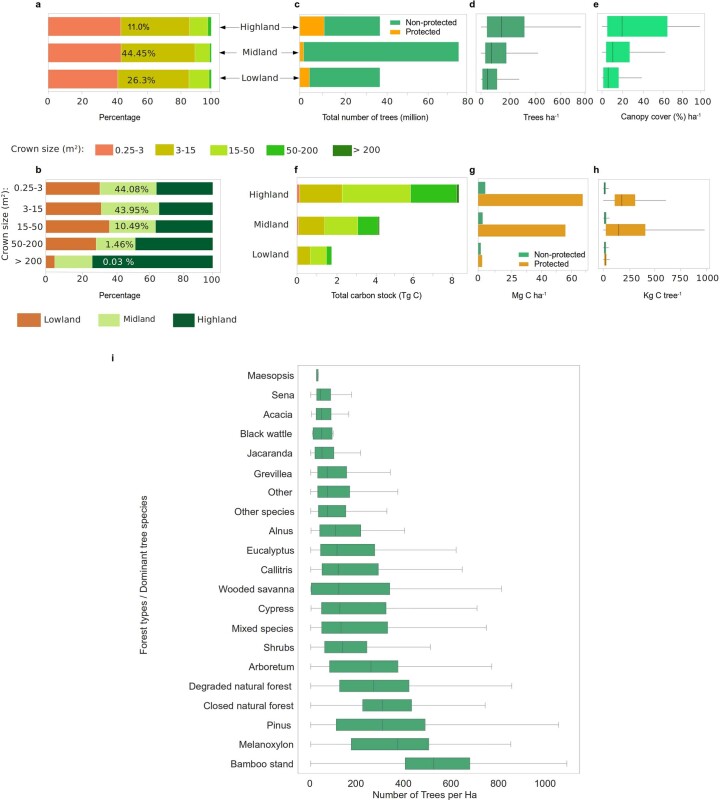
Tree properties of different landscape types. **a**, Percentage covered by different crown sizes in each landscape type. The percent number shows the contribution of the landscape type to the total area. **b**, Percentage covered by each landscape type in different crown size categories. **c**, Total count of trees by landscape type. **d**, Boxplot showing the average number of trees per ha by landscape type. **e**, Same as d but for tree cover. **f**, Total estimated carbon stock per landscape type. **g**, Barplots showing the distribution of average carbon stock per ha per landscape type, and h, boxplots showing the average carbon stock per tree per landscape type. **i**, Average tree density per tree species in Rwanda. Species data are from Rwanda national forest map of 2012^51^. In boxplots in d, e and h (from left to right), the start of the horizontal line represents the minimum value, vertical lines represents first quartile, median and third quartile values respectively, and the end of the horizontal line represents the maximum value. In i, lines from left to right represent the minimum, first quartile, median, third quartile and maximum values. Number of trees = 355,268,345.

We use locally available climate data: mean annual rainfall, mean annual temperature and elevation data (10 × 10 m^2^ resolution) to assess relationships between tree density, crown cover and environmental gradients. We also use land cover data to extract the spatial extent of plantations, forest, farmland, and urban and built-up areas for our landscape stratification. Climate data were obtained from the Rwanda Meteorological Agency as daily records from 1971 to 2017. The national forest map was manually created in 2012 using on-screen digitizing techniques over the 2008 aerial images^35^. A forest was defined as ‘a group of trees higher than 7 m and a tree cover of more than 10% or trees able to reach these thresholds in situ on a land of about 0.25 ha or more’^51^. A shrub was defined as ‘a group of perennial trees smaller than 7 m at maturity and a canopy cover of more than 10% on a land of about 0.25 ha or more’. The forest dataset was composed of 105,690 forest polygons, classified as either natural forest (closed natural forest, degraded natural forest, bamboo stand, wooded savanna and shrubland) or ‘forest plantations’ (*Eucalyptus* spp., eucalyptus; *Pinus* spp., pine; *Callitris* spp., callitris; *Cupressus* spp., cypress; *Acacia mearnsii*, black wattle; *Acacia melanoxylon*, melanoxylon; *Grevillea robusta*, grevillea; *Maesopsis eminii*, maesopsis; *Alnus acuminata*, alnus; *Jacaranda mimosifolia*, jacaranda; mixed species, mixed; and others) (Extended Data Fig. 7i). We separate shrubland from natural forest and merged it with savanna into the class ‘savannas and shrublands’. We further separated tree plantations and grouped them into *Eucalyptus* and non-*Eucalyptus* plantations. Then, a farmland map was acquired from the Rwanda Land Management and Use Authority (RLMUA)^52^ and overlaid with the 2012 forest cover map as a reference to clean the overlapping parts, under an assumption that the overlap is due to land use dynamics. Finally, a layer marking urban and built-up areas was acquired from RLMUA as well and the same preprocessing step as done for farmlands was applied. The combination of the land cover datasets resulted in our stratification scheme with six classes: natural forests, savannas and shrublands, *Eucalyptus* plantations, non-*Eucalyptus* plantations, farmland and urban and built-up.

### Mapping of individual trees using deep learning

We used the open-source framework developed by ref. ^17^ to map individual tree crowns. The framework uses a deep neural network based on the U-Net architecture^53,54^. We trained the network using 97,574 manually delineated tree crowns spread over 103 areas/bounding boxes representing the full range of biogeographical conditions found across Rwanda. To cope with the challenge of separating touching tree crowns, we used a higher weight for boundary areas between crowns, as suggested in refs. ^17,53^. Crown sizes in the predictions were found to be 27% smaller as compared to the manual delineations within the 103 training areas, due to the applied boundary weight that emphasizes gaps between tree crowns. Therefore, to calculate the real canopy cover, we extended each predicted tree crown by 27% and dissolved the touching crowns into continuous features. We counted single tree crowns for each hectare presented here as tree density and the percentage of each hectare covered by the extended tree crowns as canopy cover.

We developed a postprocessing method that separates clumped tree crowns and fills any gap inside a single crown (Extended Data Fig. 2). Our postprocessing method, which we refer to as detect centre and relabel (DCR), determines the crown centres in the model predictions assuming that tree crowns have a round shape and then relabels the model predictions on the basis of weighted distances to the identified crown centres. First, DCR performs a distance transform, computing for each pixel the Euclidean distance to the nearest pixel predicted as background. Let the transformed image be distance-transformed (DT). Then an *m* × *m* maximum filter is applied to DT, where *m* depends on the size of the smallest object to be separated. We store all pixels for which the original DT value is the same before and after max-filtering. These pixels are the instance centres as they are furthest away from the boundary and have the highest distance values within the area defined by *m*. In the case of several connected instance centres in regions where multiple connected pixels have the same distance from the background, only a single instance centre is kept. Finally, each pixel *x* predicted as a crown in the original image is assigned to its nearest instance centre, where the distance function penalizes background pixels on the connecting line between the instance centre and *x*.

### Allometry for biomass and carbon stock estimation

Generally, allometric equations define a statistical relationship between structural properties of a tree and its biomass^55,56^. In our case, we assume a relationship between the crown area and aboveground biomass (AGB), which varies between biomes^36^. Since destructive AGB measurements are rare, we established biome-specific relationships between crown diameter (CD) derived from the crown area (CD = 2√(crown area/π)) and stem diameter at breast height (DBH) (equations (3) and (6)). DBH has been shown to be highly correlated with AGB^36–40^. We then used established relationships from literature to derive AGB from DBH for savannas and shrublands (equation (4)), tree plantations (equation (5)) and natural forests (equation (7)). AGB was predicted for each tree and summed for 1 ha grids to derive AGB in the unit Mg per ha. Values were multiplied by 0.47 (refs. ^57,58^) to derive aboveground carbon (AGC). Summed numbers over land cover classes are considered as carbon stocks. The bias as reported here was calculated following the approach from ref. ^36^ reporting the relative systematic error in per cent:

1 $$\mathrm {bias} = \frac{1}{N}\mathop {\sum}\limits_{i = 1}^N {\frac{{(Y_{\mathrm {obs}} - Y_{\mathrm {pred}})}}{{Y_{\mathrm {obs}}}}}\times 100$$

The error for the evaluation with NFI data was defined by:

2 $$\mathrm{bias} = \frac{{\left| {\mathop {\sum}\nolimits_N {(Y_{\mathrm{obs}} - Y_{\mathrm{pred}})} } \right|}}{{\left| {\mathop {\sum}\nolimits_N {Y_{\mathrm{obs}}} } \right|}}$$

For trees outside natural forests, we used the database from ref. ^36^ including 10,591 field-measured trees from woodlands and savanna plus 952 samples from agroforestry landscapes in Kenya^37^ to establish a linear relationship between CD and DBH (Extended Data Fig. 3a). The Kenyan dataset is compatible with the trees in Rwanda. To ensure compatibility, the Kenya data contained open-grown trees most of which are of the same families or genus as in Rwanda grown under the same conditions, the latter factor shown to be important for generalizing^37^.

A major axis regression (average of four runs each 50% of the data) led to equation (3):

3 $${{{\mathrm{DBH}}}}_{{{{\mathrm{predicted}}}}}\,{{{\mathrm{in}}}}\,{{{\mathrm{cm}}}} = - 4.665 + 5.102 \times {{{\mathrm{CD}}}}$$

Equation (3) showed a reasonable performance with a very low bias (average of four runs on the 50% not used to establish the equation (3)): *r*² = 0.71; slope = 0.95; root mean square error (RMSE) = 6.2 cm; relative RMSE (rRMSE) = 42%; bias = 1%). We tested equation (3) on an independent dataset from Kenya consisting of 93 trees where AGB was destructively measured (Fig. 3b). The Kenyan database provides an uncommon opportunity to use destructive samples in which the carbon mass is not estimated indirectly and the relationship between crown area and carbon is direct: we do not need to invoke a second allometry to derive the dependent variable. All trees were open-grown trees in the same growing conditions as the agricultural areas of Rwanda. On these 93 trees, DBH can be predicted reasonably well from CD using equation (3) (*r*² = 0.84; slope = 0.86; RMSE = 8 cm; rRMSE = 25%; bias = 6%). We then applied an allometric equation from literature^37^ established for non-forest trees in East Africa to estimate AGB from DBH_predicted_ and compared the predicted AGB with the destructively measured AGB (*r*² = 0.81; RMSE = 511 kg; rRMSE = 55%; bias = 25%) showing an acceptable performance (Extended Data Fig. 3c) but indicating a systematic bias, which will be further tested with biome-specific field data (next section). We apply equation (4) to estimate AGB for trees outside forests in Rwanda in savannas and shrublands:

4 $${{{\mathrm{AGB}}}}_{{{{\mathrm{predicted}}}}}\,{{{\mathrm{in}}}}\,{{{\mathrm{kg}}}} = 0.091 \times {{\mathrm{DBH}}_{{\mathrm{predicted}}}}^{2.472}$$

Given the different structure of trees in farmlands, urban and built-up areas and plantations as compared to trees in natural forests and in natural non-forest areas, we used a different equation for trees in these areas. It was established in Rwanda using destructive samples from tree plantations^39^:

5 $${{{\mathrm{AGB}}}}_{{{{\mathrm{predicted}}}}}\,{{{\mathrm{in}}}}\,{{{\mathrm{kg}}}} = 0.202 \times {{\mathrm{DBH}}_{{\mathrm{predicted}}}}^{2.447}$$

A different CD–DBH relationship was established for natural forests. Here, we conducted a field campaign in December 2021 sampling 793 overstory trees in Rwanda’s protected natural forest. We measured both CD and DBH and established a logarithmic major axis regression model with a Baskerville correction^59^ between the two variables to predict DBH from CD (Extended Data Fig. 3d). We did four runs each using 50% of the data to establish equation (6) (average of the four runs) and the other 50% to test the performance also averaged over the four runs (*r*² = 0.71; slope = 0.99; RMSE = 13 cm; rRMSE = 45%; bias = 19%). Note that CD is extended by 27% to account for underestimations of touching crowns in dense forests (see previous section):

6 $$\begin{array}{l}{\mathrm{DBH}}_{{\mathrm{predicted}}}\,{\mathrm{in}}\,{\mathrm{cm}} = \left({\mathrm{exp}}\left(1.154 + 1.248 \times {\mathrm{ln}}({\mathrm{CD}} \times 1.27) \right)\right.\\\left. \times \left({\mathrm{exp}}(0.3315^2/2) \right) \right)\end{array}$$

We then used a state-of-the-art allometric equation established for tropical forests^38^ to predict AGB from DBH for natural forests in Rwanda:

7 $$\begin{array}{l}{{{\mathrm{AGB}}}}_{{{{\mathrm{predicted}}}}}\,{{{\mathrm{in}}}}\,{{{\mathrm{kg}}}} = {{{\mathrm{exp}}}}\Big[ {1.803 - 0.976{{{E}}} + 0.976\,{{{\mathrm{ln}}}}\left( \rho \right)}\\+ 2.673\;{{{\mathrm{ln}}}}\left( {{{{\mathrm{DBH}}}}} \right) - 0.0299\left[ {{{{\mathrm{ln}}}}\left( {{{\mathrm{DBH}}}} \right)} \right]^2 \Big]\end{array}$$

where *E* measures the environmental stress^38^ (a gridded layer is accessible via https://chave.ups-tlse.fr/pantropical_allometry.htm) and *ρ* is the wood density. Here, we used a fixed number (0.54), which is the average wood density for 6,161 trees from ref. ^40^, weighted according to the abundance of the species in the plots. The relative error was calculated by the quadratic mean of the intraplot and interplot variations, which is 18.2% (Extended Data Table 1b). No destructive AGB measurements were found that showed a similar CD–DBH relationship as we measured during the field trip in Rwanda’s forest. We could thus not evaluate the performance for natural forests at tree level but had to rely on plot-level comparisons (next section).

### Evaluation and uncertainties of the allometry

Biomass estimations without direct measurements of height or DBH inevitably include a relatively high level of uncertainty at tree level^38,60^. Uncertainty does not only originate from the CD to DBH conversion but also the equation converting DBH to AGB. As shown in the previous section, no strong systematic bias could be detected for the CD to DBH conversion but the evaluation of the CD-based AGB prediction with an independent dataset from destructively measured AGB revealed a bias of 25%. However, this comparison (Extended Data Fig. 3c) may not be representative for an entire country having a variety of landscapes and tree species, so a systematic propagation is unlikely. We also did not have sufficient field data to evaluate the conversions in natural forests. Here, we used data from 15 natural forest plots with 6,161 trees published by ref. ^40^ and ref. ^41^ and directly compared the summed biomass of the trees we predicted over their plots. The median measured biomass for the plots is 121 MgC ha^−1^ and we predict a median biomass of 81 MgC ha^−1^ (plot-based rRMSE = 54%; bias = 11%; bias on summed plots = 26%). The overall underestimation by our prediction is not necessarily a model bias but may be partly explained by the contribution of the understory trees, which cannot be captured by aerial images. Interestingly, our C stock estimates are in the same range of magnitude as global biomass products^43–45,61^ (Extended Data Fig. 4), indicating that overstory tree-level carbon stock assessments are possible from optical very high resolution images, even in tropical forests. Several global products overestimated biomass for non-forest areas like savannas or croplands, which is probably because they are calibrated in denser forests. The most recent products of ref. ^42^ and ref. ^61^ are much closer to the estimates from our results and the NFI. This is also seen in the grid-based correlation matrix where ref. ^42^ correlates best with our map, followed by ref. ^61^.

We further use NFI data from 2014 to measure the uncertainty of the final carbon stock estimates and evaluate if systematic differences between AGB predictions and field assessments can be found for different land cover classes (Extended Data Table 1). For the NFI data, a total of 373 plots with 2,415 trees were measured and species-specific allometric equations applied^62^. To identify systematic errors at landscape scale, we extracted averaged values for areas around the plots from our predictions and calculated statistics on averages over all plots. Interestingly, our predictions for farmlands only show a bias of 5.9%: we estimate on average 2.46 MgC ha^−1^ and the inventories measure 2.37 MgC ha^−1^ on their 150 plots. For savanna and shrublands, we estimate 4.16 MgC ha^−1^ while inventories measure 3.31 MgC ha^−1^ (bias = 18.9%). For plantations, we estimate lower values (8.16 compared to 16.79 MgC ha^−1^; bias = 52.6%). To calculate the total uncertainty on country-wide C stock estimates, we weighted the bias from the different classes according to their relative area. We estimate a total uncertainty on the carbon stock predictions of 16.9% at the national scale (Extended Data Table 1).

We found a very low bias for estimated C density in farmlands (5.9% bias) which make up most of the areas outside natural forests in Rwanda (Extended Data Table 1, Extended Data Fig. 6). The high bias for plantations can be explained by three factors: large bare areas considered part of plantations by the manual delineation of plantation areas (Extended Data Fig. 1); regular harvesting and continual thinning which keep many plantation trees young and small; and the fact that our aerial images are from 2008 while plantation trees have grown until 2014 with a few new NFI plots initiated after 2008. The bias in savannas and shrublands can be explained by the following factors: the presence of multistemed trees with large crowns such as *Acacia* spp. and *Ficus* spp. among others; the fact that a crown-based method overestimates C stocks of shrubs with a small height; and presence of shrub trees with both small height and small (multiple) stems. If tree-level based carbon stock assessments derived from crown diameter as presented here should become standard to complement national inventories, a database with sufficient samples to evaluate for systematic errors needs to be established for each biome and inventory and satellite/aerial image-based methods need to be further harmonized.

To further quantify the error propagation of the CD to DBH conversion for our application, we established four equations each randomly using 50% of the dataset and predicted the carbon stock for each tree in Rwanda with each equation. We did this separately for natural forests and trees outside natural forests. We calculated the rRMSE between the aggregated carbon stocks for each hectare. We averaged the rRMSE for each land cover class and show that the uncertainty for all classes does not exceed 5% (Extended Data Table 2a).

### Evaluation and uncertainties of tree crown mapping

We created an independent test dataset, which was never seen during training and was also not used to optimize hyperparameters. The test set consists of 6,591 manually labelled trees located in 15 random 1 ha plots (Extended Data Fig. 5). Thanks to the size of the country, the plots represent all rainfall zones and three major landscapes of the country. The plot-level comparison yielded very high correlations between the predictions and the labels and is shown in Extended Data Fig. 5. We also calculated a confusion matrix showing an overall per pixel accuracy of 96.2%, a true positive rate of 79.6% and a false positive rate of 6.8% (Extended Data Table 2b). Trees outside natural forests are easy to spot and count for the human eye, so we have confidence in the plot-based evaluation. However, it is often challenging in natural forests. Here, we used again the field measurements from 15 plots with 6,161 trees^40,41^. We find that we underestimate the total tree count by 22.6%, which may, at least partly, be explained by understory trees hidden by overstory trees and which are, therefore, not visible in our images. New field campaigns are needed to better understand and calibrate our results and possibly correct for systematic bias.

### Application and evaluation beyond Rwanda

**Extended Data Fig. 8:**
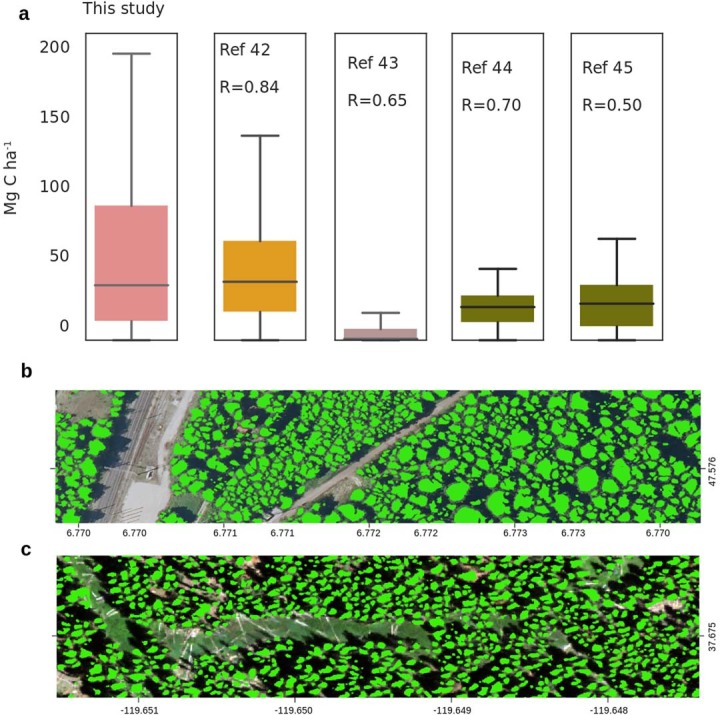
Beyond Rwanda. **a**, We took 150 (n = 150) random 1 × 1 km samples and aggregated the predicted tree-level carbon stocks and compared the results to previously published maps using two-tailored Pearson correlation. We did not include ref. ^61^ because of the coarse native resolution which did not guarantee that the same areas were compared. Note that ref. ^45^ does not report values above 85 Mg biomass per hectare, explaining the lower correlation values. P < 0.001 for all correlations. **b**, The model trained on the Rwanda aerial images was directly applied on aerial images from France (20 cm) and c, NAIP aerial images at 60 cm. In boxplots in a, lines from top to bottom represent the maximum, third quartile, median, first quartile and minimum values. Credit: photographs in **b**, USGS NAIP; **c**, IGN France.

We acquired 83 Skysat scenes at 80 cm for Tanzania, Burundi, Uganda, Rwanda and Kenya. The model trained on the 25 cm resolution aerial images of Rwanda from 2008 was directly applied on the Skysat images. Forest and non-forest areas were manually delineated to decide which allometric equation to use for the carbon stock conversion. We randomly selected 150 1 × 1 km^2^ patches and aggregated the predicted carbon density per patch and compared the results with previously published maps^42–45^. Results show that the model can directly be applied to comparable landscapes on different datasets. Note, however, that accurate carbon stock predictions need local adjustments with field data. We then tested the tree crown model transferability on aerial images from California (NAIP; 60 cm) and France (20 cm) and found that the model delivers realistic results without any local training or calibration (Extended Data Figure 8).

### Reporting summary

Further information on research design is available in the Nature Portfolio Reporting Summary linked to this article.

## Online content

Any methods, additional references, Nature Portfolio reporting summaries, source data, extended data, supplementary information, acknowledgements, peer review information; details of author contributions and competing interests; and statements of data and code availability are available at 10.1038/s41558-022-01544-w.

### Supplementary information

Supplementary Information Supplementary Figs. 1 and 2.

Reporting Summary

## Figures and Tables

**Extended Data Table 1:**
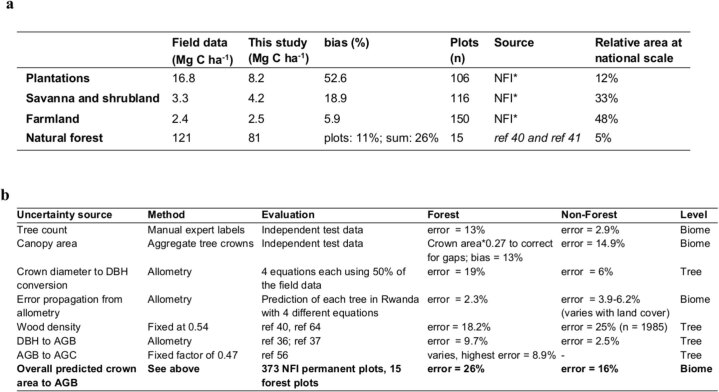
Evaluation with field data

a, We compared our tree-level carbon stock estimates aggregated to 1 ha grids with field data from different sources. The exact location of the NFI plots was unknown, so we used averages of the areas close by. The statistics were then calculated for averages per class, except for natural forests where exact plot coordinates were available, therefore, comparisons could be done per plot. We then weighted the bias according to the relative area at the national scale to derive a nation-wide uncertainty of 16.9% (NFI total C in TOF: 8.35 TgC; this study estimates 6.95 TgC). *The NFI original measurements are the aboveground biomass expressed in volume (m^3^). We have converted them in weight (metric tonnes) using the conversion rate: 1 m^3^ = 0.714 tonne (ref. ^65^) which assumes an overall average wood density of 0.714 g/cm^3^ per tree, then multiplied by the AGB-to-AGC conversion factor: 0.47 to get the C equivalent. b, Different sources of uncertainty and how we evaluated and quantified their impact. Errors at the biome level may be systematic. Errors at tree level were evaluated with equation (1), and errors at biome and plot level with equation (2)

**Extended Data Table 2:**
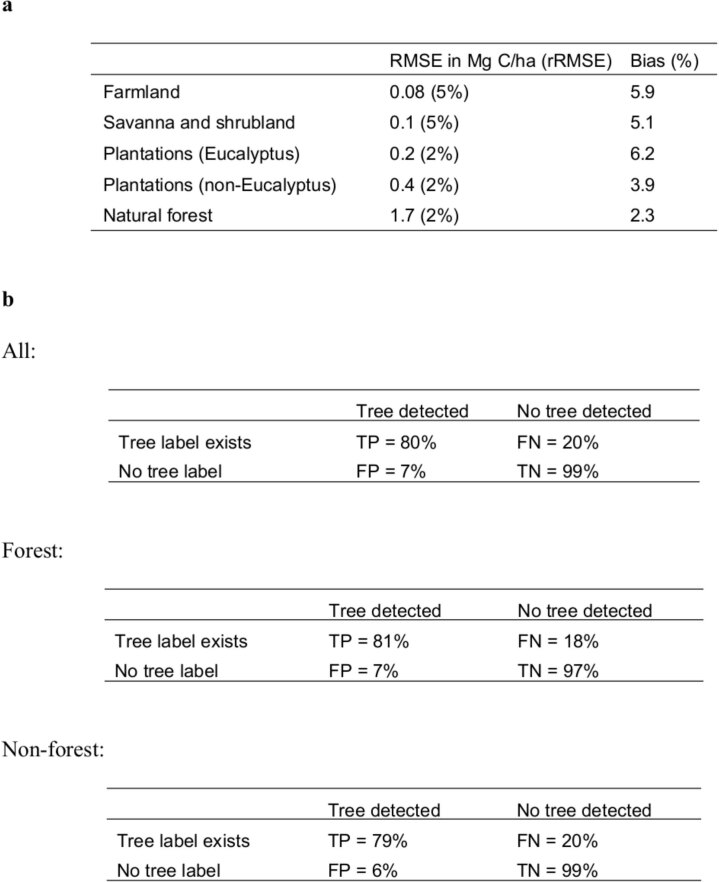
Evaluation

a, Evaluation of the crown diameter to stem diameter conversion. Four CD–DBH equations were established, each using a random subset of 50% of the data. Tree biomass was predicted with each equation and aggregated to the hectare level. We then calculated the RMSE, rRMSE and bias between the 4 predictions for each 1 ha grid. Results shown here are aggregated over land cover classes. b, Tree crown model evaluation at pixel level. Confusion matrix between predicted and 6,591 manually labelled trees. These data are independent and were not used to establish or validate the model and the results.

## Data Availability

Global tree cover maps are available at http://earthenginepartners.appspot.com/science-2013-global-forest. Climate data are freely accessible through an online application to the Rwanda Meteorological Agency via http://mis.meteorwanda.gov.rw/. Aerial images and land use and land cover data are freely available for research through formal application to the Rwanda Land Management and Use Authority at https://www.rlma.rw. Products produced in this study: tree density, tree cover, carbon stock estimates are freely accessible at 10.5281/zenodo.7118176 (ref. ^63^). The global database with tree measurements including biomass is available from J.C. Tree measurements from Kenya are available from D.S. Tree measurements from Rwanda are available from M.M.

## Code availability

The code for the tree detection framework based on U-Net is publicly available at 10.5281/zenodo.3978185. Support and more information are available from A.K. (ak@di.ku.dk or ankit.ky@gmail.com) or F.R. (flr@ign.ku.dk).
