# Supplementary material for: Nation-wide mapping of tree-level aboveground carbon stocks in Rwanda
Source: Nat Clim Chang. 2022 Dec 22;13(1):91–7. doi: 10.1038/s41558-022-01544-w (PMC9845119; doi:10.1038/s41558-022-01544-w)
Supplement: Supplementary file 1 — Supplementary Figs. 1 and 2. [file 41558_2022_1544_MOESM1_ESM.pdf]

nature climate change

Article https://doi.org/10.1038/s41558-022-01544-w

Nation-wide mapping of tree-level aboveground carbon stocks in Rwanda

In the format provided by the authors and unedited

Content:

Supplementary Figure 1
Supplementary Figure 2

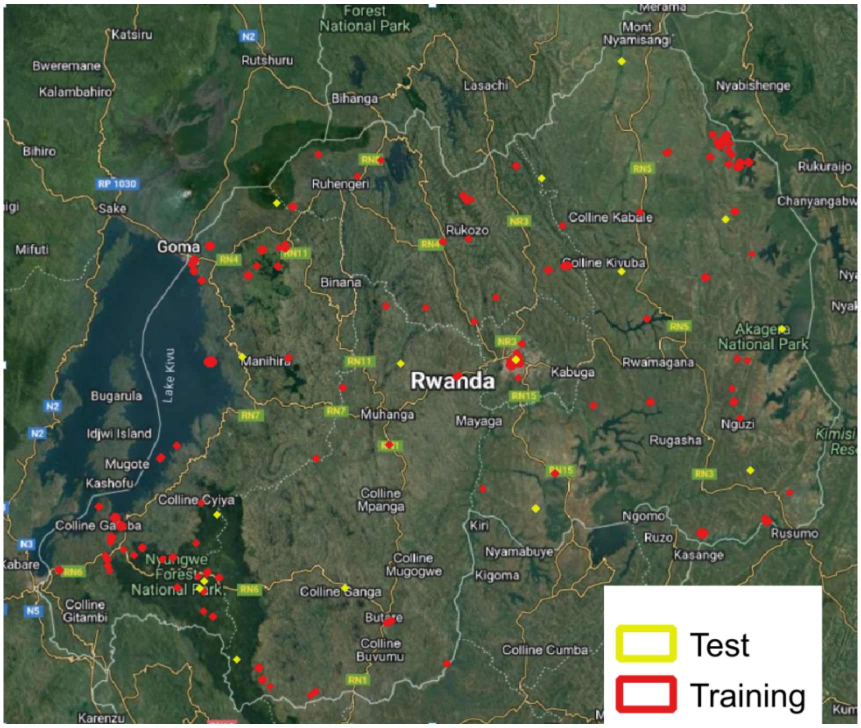

Supplementary Figure 1. Location of the training (n=103) and test areas (n=15). Background map: Google Earth satellite basemaps, Maxar 2022 http://www.earth.googl.com, accessed July, 2022.

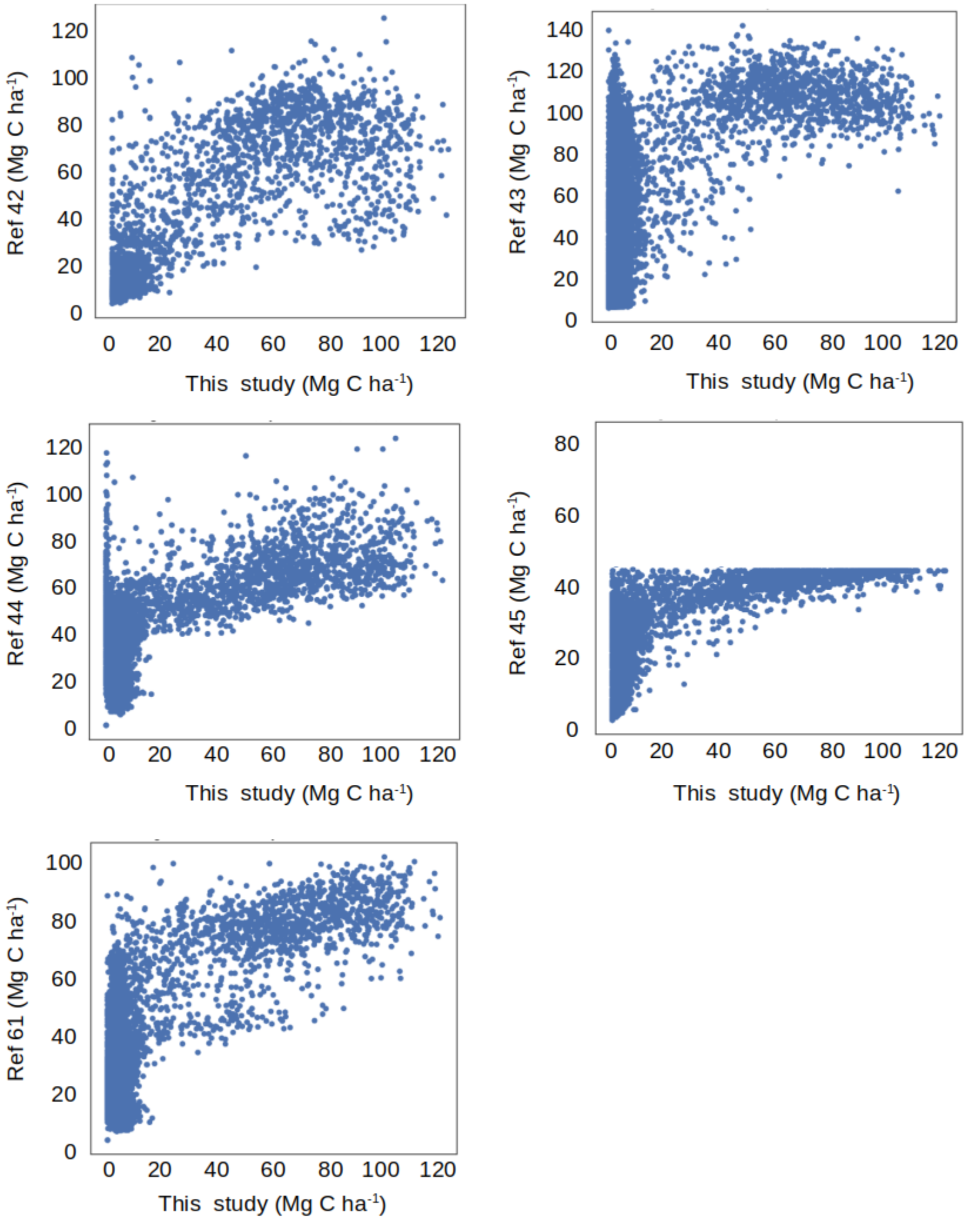

Supplementary Figure 2. Scatterplots of previously published biomass maps and the results of this study, aggregated to 1x1 km grids. N=11,227. Note that ref *45* does not report values above 85 Mg biomass per hectare.
