# Supplementary material for: Nation-wide mapping of tree-level aboveground carbon stocks in Rwanda
Source: Nat Clim Chang. 2022 Dec 22;13(1):91–7. doi: 10.1038/s41558-022-01544-w (PMC9845119; doi:10.1038/s41558-022-01544-w)
Supplement: Supplementary file 2 — Reporting Summary [file 41558_2022_1544_MOESM2_ESM.pdf]

nature research

Corresponding author(s): Martin Brandt; Maurice Mugabowindekwe

Last updated by author(s): Oct 20, 2022

Reporting Summary

Nature Research wishes to improve the reproducibility of the work that we publish. This form provides structure for consistency and transparency in reporting. For further information on Nature Research policies, see our Editorial Policies and the Editorial Policy Checklist.

Statistics

For all statistical analyses, confirm that the following items are present in the figure legend, table legend, main text, or Methods section.

n/a	Confirmed	
☐	☒	The exact sample size (*n*) for each experimental group/condition, given as a discrete number and unit of measurement
☒	☐	A statement on whether measurements were taken from distinct samples or whether the same sample was measured repeatedly
☐	☒	The statistical test(s) used AND whether they are one- or two-sided<br>*Only common tests should be described solely by name; describe more complex techniques in the Methods section.*
☒	☐	A description of all covariates tested
☒	☐	A description of any assumptions or corrections, such as tests of normality and adjustment for multiple comparisons
☐	☒	A full description of the statistical parameters including central tendency (e.g. means) or other basic estimates (e.g. regression coefficient) AND variation (e.g. standard deviation) or associated estimates of uncertainty (e.g. confidence intervals)
☒	☐	For null hypothesis testing, the test statistic (e.g. *F*, *t*, *r*) with confidence intervals, effect sizes, degrees of freedom and *P* value noted<br>*Give P values as exact values whenever suitable.*
☒	☐	For Bayesian analysis, information on the choice of priors and Markov chain Monte Carlo settings
☒	☐	For hierarchical and complex designs, identification of the appropriate level for tests and full reporting of outcomes
☒	☐	Estimates of effect sizes (e.g. Cohen's *d*, Pearson's *r*), indicating how they were calculated

Our web collection on statistics for biologists contains articles on many of the points above.

Software and code

Policy information about availability of computer code

Data collection	The aerial image data were provided by the Centre for Geographic Information Systems and Remote Sensing of University of Rwanda. There was no special software involved
Data analysis	Only free and open source software was used for data analysis: Python (3.8), RStudio (1.4), QGIS (3.22), GDAL tools (3.1). The deep learning code was written in Python (3.8) using Tensoflow (2.5). The clumped trees separation code was written in Python (3.8)

For manuscripts utilizing custom algorithms or software that are central to the research but not yet described in published literature, software must be made available to editors and reviewers. We strongly encourage code deposition in a community repository (e.g. GitHub). See the Nature Research guidelines for submitting code & software for further information.

Data

Policy information about availability of data

All manuscripts must include a data availability statement. This statement should provide the following information, where applicable:

- Accession codes, unique identifiers, or web links for publicly available datasets
- A list of figures that have associated raw data
- A description of any restrictions on data availability

Global tree cover maps are available at http://earthenginepartners.appspot.com/science-2013-global-forest. Climate data are freely accessible through an online application to the Rwanda Meteorological Agency via http://mis.meteorwanda.gov.rw/. Aerial images, and land use and land cover data are freely available for research through formal application to the Rwanda Land Management and Use Authority: https://www.rlma.rw. Products produced in this study: tree density, tree cover, carbon stock estimates are freely accessible at https://doi.org/10.5281/zenodo.7118176 (ref 65). The global database with tree measurements including biomass is available from J.C.. Tree measurements from Kenya are available from D.S.. Tree measurements from Rwanda are available from M.M.. Any more relevant data can be availed upon reasonable request addressed to the corresponding authors.

Field-specific reporting

Please select the one below that is the best fit for your research. If you are not sure, read the appropriate sections before making your selection.

☐ Life sciences ☐ Behavioural & social sciences ☒ Ecological, evolutionary & environmental sciences

For a reference copy of the document with all sections, see nature.com/documents/nr-reporting-summary-flat.pdf

Ecological, evolutionary & environmental sciences study design

All studies must disclose on these points even when the disclosure is negative.

Study description	This study uses 198 aerial images at 0.25 m2 resolution. We used a deep learning algorithm trained on 97,574 hand-lebelled tree crowns to map 355,268,345 trees with a crown size larger than 0.25 square meters both inside and outside forests in Rwanda, a country located in East Africa, spanning a wide range of African landscapes: natural forests including tropical montane rainforest, savannas, shrublands, tree plantations including young plantations and coppices, agroforestry trees in farmlands, and isolated trees. Furthermore, we apply state-of-the art allometric equations to estimate and map carbon stock of every mapped individual tree at the national scale.
Research sample	The study is a nation-wide mapping of individual tree carbon stocks, where over 355 million trees were mapped, and charecterised in terms of their crown size and carbon stock all over Rwanda: a country with surface area of more than 25 thousand square kilomeres, excluding an area of about 4,000 square kilometers where 0.25 m resolution aerial images were not available.
Sampling strategy	We did not do a specific sampling, because all trees and shrubs are included in the study. However, we have excluded any woody plant with a crown size below 0.25 m2, a threshold set based on the visual inspection of the aerial images, as trees of this size and above were still clearly visible
Data collection	We use publicly available aerial images of Rwanda at 0.25x0.25 m resolution, collected in June - August of 2008 and 2009. The images were acquired from 3000 meter altitude above ground level, initially with a mean ground resolution of 0.22x0.22 m pixel size, using a Vexcel UltraCam-X aerial digital photography camera. They include a red, green, and blue band stored under 8 bit unsigned integer format. The aerial images cover 96% of the country, and the remaining 4% was filled by satellite images from WorldView-2, Ikonos, Spot, and QuickBird satellite sensors which are part of the publicly available dataset. For the carbon stocks estimation, we conducted a field campaign in December 2021 and measured 793 trees in the natural forest, and also used previously published 10,591 measurements of pan-tropical non-forest trees, as well as previously published 952 non forest trees from Kenya. We validated the results with data from the Rwanda National Forest Inventory (NFI) from 2013/2014 collected in 373 NFI permanent plots with 2,415 trees, and previously published 6161 measurements of afromontane rainforests in Rwanda.
Timing and spatial scale	The aerial images are from June-August 2008 and 2009, and cover the extent: 28.86,-2.84 : 30.90,-1.05 of Rwanda in East Africa.
Data exclusions	Aerial images were taken only during dry season of 2008 and 2009 because it is time when the sky is clear and features on the ground are very visible for the air-borne or space-borne sensor. However, 4% of the country (about 100,000 km2) was not covered by the aerial images due to permanent clouds or trans-boundary security issues during the images acquisition, which made it imperative to opt for image fusion approach using WorldView-2, Ikonos, Spot, and QuickBird satellite images. However, there is an area about 4,000 km2 around the location (longitude: 29.62, latitude: -1.95) where fusion has used a 2.5-m resolution images from Spot satellite sensor, and trees were not accurately visible. This area was excluded from further analysis.
Reproducibility	Data were processed 3 times at different time intervals leading to the same results
Randomization	NA, we have used all 355 milllion trees with no sampling
Blinding	NA, we have used all 355 million trees with no sampling

Did the study involve field work? ☒ Yes ☐ No

Field work, collection and transport

Field conditions	Fieldwork was conducted in December 2021. The conditions were humid with temperatures around 20oC
Location	The data collection was done in South-western part of Rwanda, in the tropical montane forest located in a protected area of Nyungwe National Park (29.31, -2.51)
Access & import/export	NA, the data were noted digitally
Disturbance	Measuring the tree dimensions (crown diameter, tree height, diameter at breast height, tree location) did not involve disturbances. However, since the field collection area is a protected area with a natural forest, we had to work with forest rangers to guide our field work and advise on accessible plot locations. This way, we followed existing trails and ensured not to do any destructive sampling or vegetation clearing for new trails. To abide by the conditions of the area, our sample plots were limited to locations within 200 m from the existing major roads within the forest

Reporting for specific materials, systems and methods

We require information from authors about some types of materials, experimental systems and methods used in many studies. Here, indicate whether each material, system or method listed is relevant to your study. If you are not sure if a list item applies to your research, read the appropriate section before selecting a response.

Materials & experimental systems

n/a	Involved in the study
☒	☐ Antibodies
☒	☐ Eukaryotic cell lines
☒	☐ Palaeontology and archaeology
☒	☐ Animals and other organisms
☒	☐ Human research participants
☒	☐ Clinical data
☒	☐ Dual use research of concern

Methods

n/a	Involved in the study
☒	☐ ChIP-seq
☒	☐ Flow cytometry
☒	☐ MRI-based neuroimaging
